# Correction: CCR7 provides localized access to IL-2 and defines homeostatically distinct regulatory T cell subsets

**DOI:** 10.1084/jem.2013114207032019c

**Published:** 2019-07-10

**Authors:** Kate S. Smigiel, Elizabeth Richards, Shivani Srivastava, Kerri R. Thomas, Jan C. Dudda, Kimberly D. Klonowski, Daniel J. Campbell

Vol. 211, No. 1, January 13, 2014. 10.1084/jem.20131142.

The authors regret that in the original version of Fig. 3 A, the axes labels in the flow cytometry plots were incorrect. The revised panel appears below with corrected labels “Ki-67” and “pStat5.” The authors apologize for the mistake made during figure preparation.

**Figure:**
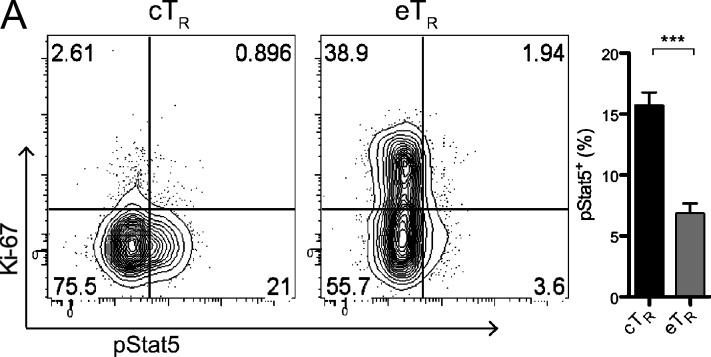


The online HTML and PDF versions of this paper have been corrected. The error remains only in the print version.

